# *TaRac6* Is a Potential Susceptibility Factor by Regulating the ROS Burst Negatively in the Wheat–*Puccinia striiformis* f. sp. *tritici* Interaction

**DOI:** 10.3389/fpls.2020.00716

**Published:** 2020-06-30

**Authors:** Qiong Zhang, Xinmei Zhang, Rui Zhuang, Zetong Wei, Weixue Shu, Xiaojie Wang, Zhensheng Kang

**Affiliations:** ^1^State Key Laboratory of Crop Stress Biology for Arid Areas, College of Plant Protection, Northwest A&F University, Yangling, China; ^2^College of Life Sciences, Northwest A&F University, Yangling, China

**Keywords:** TaRac6, wheat, *Puccinia striiformis* f. sp. *tritici*, reactive oxygen species, susceptibility factor

## Abstract

Rac/Rop proteins play important roles in the regulation of cell growth and plant defense responses. However, the function of Rac/Rop proteins in wheat remains largely unknown. In this study, a small G protein gene, designated as *TaRac6*, was characterized from wheat (*Triticum aestivum*) in response to *Puccinia striiformis* f. sp. *tritici* (*Pst*) and was found to be highly homologous to the Rac proteins identified in other plant species. Transient expression analyses of the TaRac6-GFP fusion protein in *Nicotiana benthamiana* leaves showed that TaRac6 was localized in the whole cell. Furthermore, transient expression of TaRac6 inhibited Bax-triggered plant cell death (PCD) in *N. benthamiana*. Transcript accumulation of *TaRac6* was increased at 24 h post-inoculation (hpi) in the compatible interaction between wheat and *Pst*, while it was not induced in an incompatible interaction. More importantly, silencing of *TaRac6* by virus induced gene silencing (VIGS) enhanced the resistance of wheat (Suwon 11) to *Pst* (CYR31) by producing fewer uredinia. Histological observations revealed that the hypha growth of *Pst* was markedly inhibited along with more H_2_O_2_ generated in the *TaRac6*-silenced leaves in response to *Pst*. Moreover, transcript levels of *TaCAT* were significantly down-regulated, while those of *TaSOD* and *TaNOX* were significantly up-regulated. These results suggest that *TaRac6* functions as a potential susceptibility factor, which negatively regulate the reactive oxygen species (ROS) burst in the wheat–*Pst* interaction.

## Introduction

Small GTP-binding proteins are proteins that have a molecular weight of 20–40 kd. They constitute a superfamily with five families—Ras, Ran, Rab, Rho, and Arf—which includes more than 100 members (Takai et al., 2001). The Rho family in animals is further divided into three subfamilies: Rho, Rac, and CDC42. However, because the Rho family in plants more closely resembles the Rac subfamily in animals, they are also called Rac-like or Rop-like (Rac/Rop) proteins (Winge et al., 2000).

The Rac/Rop protein family contains five highly conserved G-boxes and a C-terminal motif (Wennerberg et al., 2005). One G-box is the binding region of downstream effectors. The function of the other four G-boxes is to bind GTP/GDP and to hydrolyze GTP to GDP. The C-terminal motifs are related to the function of GTPase and the subcellular localization (Williams, 2003). Based on its C-terminal motifs, Rac/Rop proteins may be divided into two types (Winge et al., 2000): Type I have a conserved CaaL motif (a: aliphatic amino acid), while Type II lack the CaaL motif (Lavy et al., 2002) but have a cysteine domain to the membrane (Kawano et al., 2014). All type-I Rac/Rop proteins are prenylated (Lavy et al., 2002). Prenylation is required for membrane attachment and function of type I Rops, while type II Rops with the cysteine domain are attached to the plasma membrane by S-acylation. The prenylation of Rops determines their stable distribution between the plasma membrane and cytoplasm but has little effect on the dynamics of membrane interaction. In addition, the prenylation of type I Rops has only a small effect on ROP function. The mechanism of type II ROP S-acylation and membrane attachment is unique to plants and likely responsible for the viability of plants in the absence of CaaL prenylation activity. Type I ROPs affect the cell structure, primarily on the adaxial side, while type II ROPs induce a novel cell division phenotype (Sorek et al., 2011).

There are two states of Rac/Rop: the GTP-bound state Rac/Rop and GDP-bound state Rac/Rop, with the former being active and the latter being inactive (Vetter and Wittinghofer, 2001). GTPase-activating proteins (GAPs) reconvert the active Rac/Rop to an inactive state by promoting GTPase activity. The guanine nucleotide dissociation inhibitor (GDI) inhibits GDP-bound to GTP-bound, and guanine nucleotide exchange factors (GEFs) release GDP from Rac/Rop and bind Rac/Rop to GTP. The active Rac/Rop are able to interact with downstream effectors to function.

The Rac/Rop family is an important signal transduction regulator in plants, participating in various key life processes, including plant cell polarity, cell growth, morphological development, cytoplasmic division, signal transduction of hormones, and resistance to adversity (Schiene et al., 2000; Vernoud et al., 2003; Berken, 2006; Kawano et al., 2010a, b; Kawano and Shimamoto, 2013). However, the functioning of Rac/Rop family members in the interaction between plants and pathogens are still largely unknown. In rice (*Oryza sativa*), seven Rac/Rop family genes have been isolated (Miki et al., 2005). Among them, *OsRac1* plays a positive role in blast resistance but overexpressed transgenic plants of *OsRac4*, *OsRac5*, and *OsRac6* showed greater susceptibility to rice blast, whereas *OsRac3* and *OsRac7* may not participate in plant disease responses (Jung et al., 2006; Chen et al., 2010). OsRac1 contributes to disease resistance by regulating reactive oxygen species (ROS) and the biosynthesis of chitin and lignin (Wong et al., 2004; Kawasaki et al., 2006; Akamatsu et al., 2013). Additionally, several proteins, such as OsMAPK6, CERK1, GEF1, and SPL11, were found to be associated with Rac/Rop proteins participating in the interaction between plants and their pathogens (Lieberherr et al., 2005; Akamatsu et al., 2013; Liu et al., 2015). In barley (*Hordeum vulgare*), six Rac/Rop family genes were isolated, of which *HvRacB* was confirmed to be able to promote the susceptibility of barley to *Blumeria graminis* f. sp. *hordei* (*Bgh*) (Schultheiss et al., 2002, 2003). Furthermore, HvRacB was shown to affect barley’s resistance to *Bgh* by modulating the reorganization of actin (Opalski et al., 2005). Thus, different members of the same Rac/Rop family can play distinct roles in shaping how plants respond to pathogenic attacks and infection. Hence, it is of great significance to explore the mechanisms underpinning the Rac/Rop family genes’ involvement in plant responses to pathogens.

Wheat stripe rust, caused by *Puccinia striiformis* f. sp. *tritici* (*Pst*), is among the most devastating diseases afflicting wheat (Chen et al., 2014), having become one the most important biotic problems threatening wheat production worldwide (Schwessinger, 2017). A better understanding of host–pathogen interactions will lay a theoretical foundation to formulate new strategies for the sustainable control of stripe rust. Analysis of cDNA library data revealed a Rac/Rop homologous gene in wheat that was up regulated in a compatible interaction (Ma et al., 2009). Yet, the function of this Rac/Rop gene in wheat’s response to *Pst* is still unknown. In this study, we report on this Rac/Rop family gene, designated as *TaRac6*, which was located to the whole cell and inhibited cell death induced by Bax. The function of *TaRac6* was further analyzed using VIGS (virus induced gene silencing), which demonstrated that *TaRac6* could regulate the resistance of wheat to *Pst* negatively by affecting the ROS burst. These results lay a foundation to explore the functioning of plant Rac/Rop proteins under pathogen infection.

## Materials and Methods

### Preparation of Plant Materials, Wheat Stripe Rust and Bacterial

Wheat and tobacco (*Nicotiana benthamiana*) plants were planted at 16°C and 23°C, respectively, under 60% relative humidity. The *Pst* isolates CYR31 and CYR23 were cultured on wheat cultivars ‘Suwon11’ and ‘Mingxian169,’ respectively (Kang et al., 2002). The *Escherichia coli* strain JM109 was cultured in Luria-Bertani (LB) culture medium overnight at 37°C in the dark. The *Agrobacterium tumefaciens* strain GV3101 was cultured in LB at 30°C in the dark for 1–2 days.

### Sequence Analysis of TaRac6

The protein features were predicted in NCBI^1^. Protein molecular weight was predicted by ExPASy^2^. SignalP 4.1^3^ was used to predict protein signal peptide. TMHMM Server v. 2.0^4^ was used to predict transmembrane helices in proteins. PSORT^5^ was used to predict subcellular localization. cNLS Mapper (nls-mapper.iab.keio.ac.jp/cgi-bin/NLS_Mapper_form.cgi#opennewwindow) was used to predict nuclear location signal. The software DNAMAN6.0 was used to align multiple sequences. The Phylogenetic tree was produced with the MEGA5 using the neighbor-joining approach. The fragment used for VIGS was aligned with the whole genome information of wheat^6^ and *Pst*^7^ to ensure sequence specificity.

### Plasmid Construction

Primers used for plasmid construction are listed in Supplementary Table S1. The ORF sequence of *TaRac6* was cloned into the pCAMBIA-1302 and pBinGFP2 vectors to verify its subcellular localization. pCAMBIA-1302 vector was used to express GFP at the C-terminus of TaRac6 (TaRac6-GFP^C^) and pBinGFP2 at the N-terminus (GFP^N^-TaRac6). To silence *TaRac6*, a specific 183-bp fragment containing a 13-bp untranslated region and a 170-bp fragment of a translated region was constructed into the BSMV-γ vector. To overexpress *TaRac6* in *N. benthamiana*, the ORF sequence was inserted into the PVX vector pGR106.

### RNA Extraction and Quantitative RT-PCR

The fresh urediniospores of CYR23 (incompatible interaction) and CYR31 (compatible interaction) were inoculated on the first leaves of 7-day-old wheat seedlings (Suwon11) with an inoculation needle. After inoculation, wheat seedlings were cultured in dark for 24 h with 100% humidity, and then transferred to a greenhouse at 15°C with a 16 h photoperiod. The leaves inoculated with CYR23 and CYR31 were sampled at 0, 12, 24, 48, 72, and 120 h post-inoculation (hpi), respectively. Total RNA from each sample was extracted using the MiniBEST Universal RNA Extraction Kit (TaKaRa, Kusatsu, Japan). The quality of obtained RNA was checked by electrophoresis. As described by Feng et al. (2011), first-strand cDNA was synthesized using Oligo dT Primer.

The primers used for the qRT-PCR can be found in Supplementary Table S1. Elongation factor 1α (EF-1α) of wheat was selected as the inner reference gene (Ling et al., 2007). The procedure of qRT-PCR followed that of Feng et al. (2011). The results were analyzed using the 2^–ΔΔCT^ method (Livak and Schmittgen, 2012), with three independent biological replicates.

### Transient Expression Assays for Subcellular Localization

GV3101 carrying pCAMBIA-1302-TaRac6-GFP^C^, pCAMBIA-1302-GFP, pBinGFP2-GFP^N^-TaRac6 or 35S-mCherry plasmids were cultured in LB (50 μg/mL kanamycin and 50 μg/mL rifampicin) for 1–2 days. The cells were collected and suspended as described by Zhao et al. (2018). GFP, GFP^N^-TaRac6, TaRac6-GFP^C^ of *A. tumefaciens* were co-injected into leaves of tobacco plants 4–6 weeks old with mCherry, respectively. Two days later plant tissue samples were harvested. The GFP images were taken under a LSM510 Confocal Microscope (Zeiss, Germany) with 488 nm laser lines. The mCherry images were taken under a LSM510 Confocal Microscope (Zeiss, Germany) with 584 nm laser lines.

The expression of TaRac6-GFP^C^ and GFP^N^-TaRac6 were further confirmed by western blot. The total proteins of injected tobacco leaves were extracted using the Native lysis buffer (Solarbio, Beijing, China). Specifically, 10 μL PMSF (100 mM) and 10 μL protease inhibitor cocktail (EDTA-Free, 100 × in DMSO) were added per ml of lysate. The extraction of total protein and the western blot procedure used are described in Zhao et al. (2018).

### Inhibition Assay of PCD Induced by Bax

The pGR106-TaRac6, pGR106-eGFP (negative control), and pGR106-Avr1b (positive control, Dou et al., 2008) were respectively transformed into GV3101 (Hellens et al., 2000). Details on the treatment of the positive transformant can be found in Zhao et al. (2018). The *A. tumefaciens* cell suspensions of pGR106-TaRac6, pGR106-eGFP, and pGR106-Avr1b were injected separately into *N. benthamiana* leaves using sterile syringes. Then, 24 h later, the agrobacterium suspension containing the Bax gene was injected again at the same location. The tobacco leaves were sampled at 3 days post inoculation (dpi). The total RNA and cDNA of all samples at 3 dpi with Bax were obtained using the procedural methods described above. qRT-PCR was used to detect the transcription levels of *N. benthamiana* defense-related genes (*PR1*α, *PR2*, and *PR5*). The *N. benthamiana* housekeeping gene *NbActin* was selected as the inner reference gene. The results were analyzed by the 2^–ΔΔCT^ method (Livak and Schmittgen, 2012), using three independent biological replicates. Symptoms were observed 5–7 days later. Leaves were decolorized by ethanol/glacial acetic acid (v/v, 1:1).

### BSMV-Mediated *TaRac6* Gene Silencing

The VIGS (virus induced gene silencing) system was implemented as described by Holzberg et al. (2002). ‘Suwon11’ was the cultivar used for the experiment. To silence TaRac6, BSMV: α + β + γ-TaRac6 was used to inoculate wheat seedlings. BSMV: α + β + γ was used as the control. About 30 seedlings were inoculated with each treatment. Ten days after virus inoculation, fresh CYR31 urediniospores were inoculated onto the fourth leaf. The wheat seedlings were cultured as described by Zhao et al. (2018). Their fourth leaves were sampled at 24 hpi and 48 hpi, to detect the gene-silencing efficiency and to observe the hyphae lengths and H_2_O_2_ accumulation at the histological level. The RNA of the fourth leaves inoculated with BSMV: α + β + γ and BSMV: α + β + γ-TaRac6 were isolated, and the qRT-PCR was used to assess the silencing efficiency and expression of the *TaCAT*, *TaSOD* and *TaNOX* genes. Cytological analyses of *Pst* growth and the host response in the control and *TaRac6*-silenced wheat plants were carried out as described by Zhao et al. (2018). Thirty-five infection sites from three leaves per treatment were used to calculate the hyphal length and H_2_O_2_ accumulation. Only the infected sites with substomatal vesicles under the stomata were considered to be successfully infected. Wheat germ agglutinin conjugated to Alexa Fluor 488 (Invitrogen, Carlsbad, CA, United States) was used to stain the *Pst* infection structures as described in Ayliffe et al. (2011). The length of hyphae and accumulation of H_2_O_2_ were each observed under an Olympus BX-51 microscope. The wheat phenotypes were observed 14 days after the *Pst* inoculation (dpi). For each treatment, six inoculated leaves were used to observe the phenotype. The phenotype was quantified by calculating the uredinium number within 1 cm^2^ area for one leaf. To avoid bias among the leaf samples, test points were randomly selected from the six treated plants. To estimate changes in the fungal biomass, DNA quantification of the single-copy target genes *PsEF1* (from *Pst*) and *TaEF1* (from wheat) was further measured using qRT-PCR as previously described (Panwar et al., 2013; Liu et al., 2016). Three independent biological replicates were performed.

## Results

### Sequence Analysis of TaRac6

**FIGURE 1:**
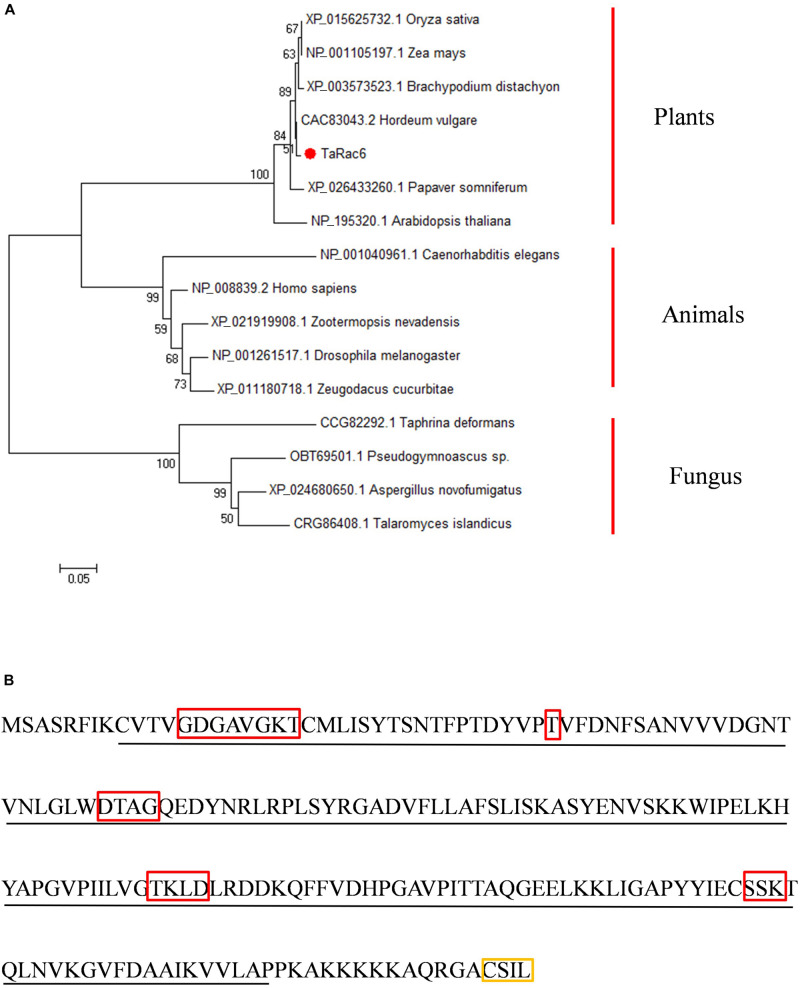
Amino acid sequences analysis and characterization. **(A)** The Phylogenetic tree of TaRac6 and RAC/ROP proteins from various eukaryotes was carried out using the MEGA5 by neighbor-joining approach. Branches are labeled with GenBank accession numbers and the corresponding name of each eukaryotic species. XP_015625732.1 (OsRac6), NP_001105197.1 (ZmRop9), XP_003573523.1 (BdRac6), CAC83043.2 (HvRopB), XP_026433260.1 (PsRac6), NP_195320.1 (AtRac6), NP_001040961.1 (CeRac2), NP_008839.2 (HsRac1), XP_021919908.1 (ZnRac1), NP_001261517.1 (DmRac2), XP_011180718.1 (ZcRac1), and CCG82292.1 (TdRho2). **(B)** Conserved domain of the *TaRac6* protein. The red boxes indicate G1–G5 boxes. The yellow box indicates the CxxL motif. The sequence underlined with a black line indicates the Rho domain.

An up-regulated transcript in the cDNA library of the compatible interaction between wheat and *Pst* was isolated (Ma et al., 2009), and designated as *TaRac6* based on the Blast results in NCBI (see foot note 1). BlastN analyses in the *Triticum aestivum* genome sequence showed that there are three copies of this gene in the wheat genome, located on 6A, 6B, and 6D. The cDNA sequence of the three copies obtained in Suwon11 are highly similar (Supplementary Figure S1) and encodes the same proteins (Supplementary Figure S2). The three copies of *TaRac6* encode the same 197 amino acids, which showed high homology with the Rac/Rop proteins from other plants (Supplementary Figure S3). TaRac6 has no signal peptide or transmembrane domain predicted by the SignalP 4.1 and TMHMM Server. It was predicted to be located in the plasma membrane, cytoplasm, and Chloroplast and has a nuclear localization signal predicted by the PSORT and cNLS Mapper. The protein features analysis indicated that TaRac6 contained a Rop-like domain (Supplementary Figure S4). Phylogenetic analysis indicated that *TaRac6* and other plant Rho-related GTPases clustered together (Figure 1A). As a protein in the Rac/Rop GTPase family, TaRac6 contains five G boxes and a CxxL motif, which is the typical motif of the Rac/Rop protein belonging to Type I (Figure 1B).

### TaRac6 Is Localized in Plasma Membrane, Cytoplasm, and Nucleus

**FIGURE 2:**
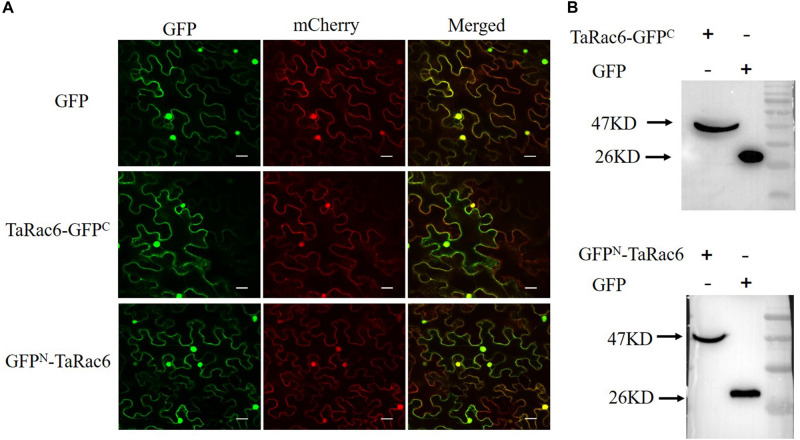
Subcellular localization of TaRac6 in tobacco leaves. **(A)** The green fluorescent protein (GFP), TaRac6-GFP^C^, and the GFP^N^-TaRac6 fusion protein were transiently expressed in *Nicotiana benthamiana* with mCherry, respectively. Signals of both were detected throughout the cell. **(B)** Western blot was used to analyze the GFP, TaRac6-GFP^C^, and GFP^N^-TaRac6 fusion protein. Bars = 20 μm.

The control GFP, the TaRac6-GFP^C^, or the GFP^N^-TaRac6 were transiently expressed in tobacco leaves with 35S-mCherry, respectively. The fluorescence of TaRac6-GFP^C^ was observed in the plasma membrane, cytoplasm, and nuclear region of *N. benthamiana.* Similarly, the signal of TaRac6-GFP^N^ was also detected in the whole cell of *N. benthamiana* (Figure 2A). Western blot assays indicated that the TaRac6-GFP^C^ and TaRac6-GFP^N^ fusion proteins were successfully expressed in *N. benthamiana* (Figure 2B).

### Transient Expression of TaRac6 Inhibits Cell Death in Tobacco

**FIGURE 3:**
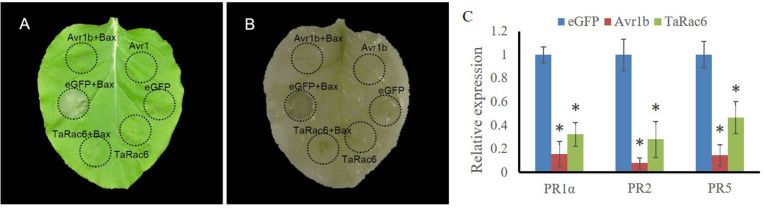
Transient expression of TaRac6 inhibited Bax-induced plant cell death in tobacco. **(A)** Tobacco leaves were injected with *Agrobacterium tumefaciens* cells containing pGR106-eGFP (negative control), pGR106-TaRac6, or pGR106-Avr1b (positive control); 24 h later, *A. tumefaciens* cells containing Bax were injected into the tobacco leaves. Photos were taken 5 days later. **(B)** Leaves were decolorized by ethanol/glacial acetic acid (1:1, v/v). **(C)** Transcription level changes of *N. benthamiana* defense-related genes *PR1*α, *PR2*, and *PR5* in tobacco leaves when Bax was co-expressed with pGR106-eGFP, pGR106-TaRac6, or pGR106-Avr1b at 3dpi. *NbActin* was selected as the inner reference gene. Three independent biological replications were performed to calculate each of the mean values. Vertical bars represent the standard deviation. *P < 0.05.

Cell death is associated with plant resistance to invasion and spread by pathogens (Van Doorn et al., 2011). Bax is a death-promoting member of the Bcl-2 family of proteins which trigger cell death when expressed in plants (Lacomme and Santa Cruz, 1999). Bax-triggered cell death has similar physiological characteristics to plant hypersensitive responses (Lacomme and Santa Cruz, 1999). To determine whether TaRac6 could induce cell death or inhibit Bax-induced cell death to affect plant defense response, TaRac6 was transiently overexpressed in tobacco leaves with the Bax system. When Bax was expressed in tobacco leaves, the cells showed obvious necrosis. By contrast, cell death induced by Bax was starkly inhibited when TaRac6 and Bax were co-expressed. However, no cell death was observed in the leaves injected with Avr1b, which served as the positive control. The transcription levels of *PR1*α, *PR2, and PR5* were reduced in tobacco leaves when Bax was co-expressed with pGR106-TaRac6 or pGR106-Avr1b, compared to pGR106-eGFP (Figure 3). These results indicated that TaRac6 could play an important role in inhibiting cell death.

### TaRac6 Is Highly Expressed in the Compatible Wheat–Pst Interaction

**FIGURE 4:**
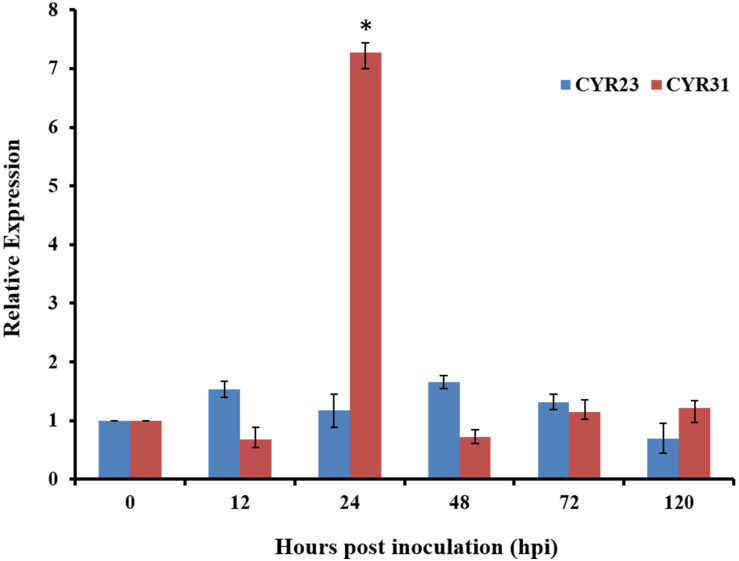
Transcript profiles of *TaRac6* in wheat inoculated with *Pst*. Expression pattern analyses of *TaRac6* during the different developing stages of the incompatible interaction (CYR23) and compatible interaction (CYR31) were calculated using the 2^–ΔΔCT^ method. Three independent biological replications were performed to calculate each of the mean values. The wheat gene *TaEF-1*α was used to normalize the qRT-PCR data. Vertical bars represent the standard deviation. *P < 0.05.

To determine whether *TaRac6* participate in wheat-*Pst* interactions, qRT-PCR was used to detect the expression of *TaRac6* in the compatible and incompatible interaction of wheat–*Pst*. The expression of *TaRac6* was up-regulated in the compatible interaction of wheat–*Pst* (CYR31). The transcript level of *TaRac6* at 24 hpi was approximately 7.2-fold that of the control (0 hpi). However, the transcript level of *TaRac6* was almost unchanged in the incompatible interaction (Figure 4). The result indicated that *TaRac6* played an important role in the compatible interaction between wheat and *Pst*.

### Silencing BSMV-TaRac6 Increased the Resistance of Wheat

**FIGURE 5:**
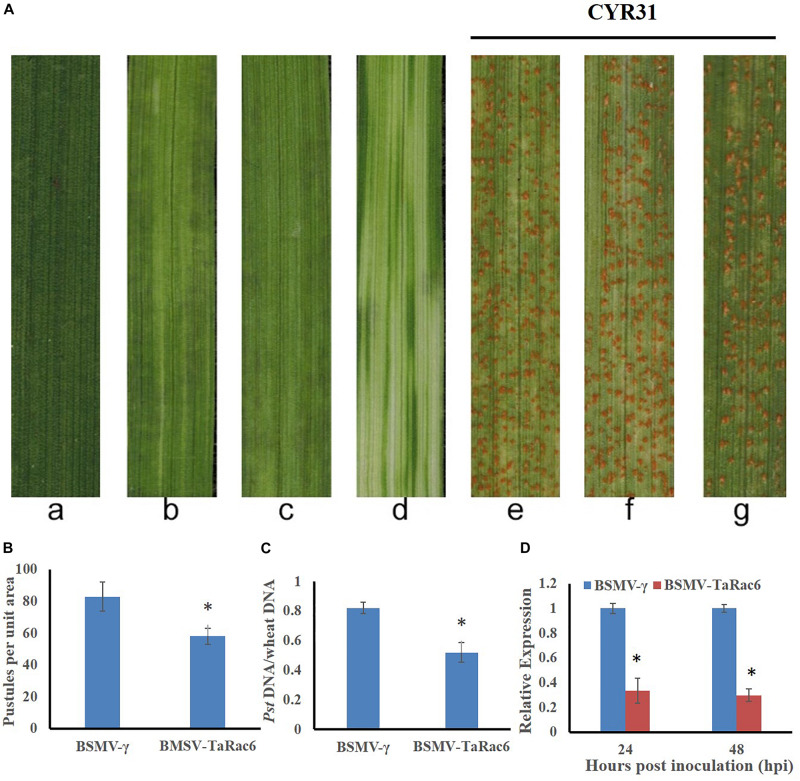
Functions of *TaRac6* silencing by virus induced gene silencing (VIGS) in the compatible interaction between wheat (Suwon 11) and stripe rust pathogen (CYR31). **(A)** Phenotypes of the fourth leaves inoculated with the FES-buffer **(a)**, BSMV-γ **(b)**, BSMV-*TaRac6***(c)**, or BSMV-PDS **(d)** at 12 days post-virus treatment. Phenotypes of the fourth leaves inoculated with CYR31 at 14-day post inoculation that had been pre-inoculated with the FES-buffer **(e)**, BSMV-γ **(f)**, or BSMV-*TaRac6* **(g)**. **(B)** Quantification of the uredinium density at 14 dpi with *Pst*. Means and standard deviations were calculated from three independent replicates. Six treated leaves were used to calculate the uredinium number per replicate. ^∗^*P* < 0.05. **(C)** Fungal biomass was measured with total genomic DNA extracted from control and TaRac6-silenced wheat plants using qRT-PCR. Means and standard deviations were calculated from three independent replicates. Samples were taken at 14-day post inoculation with *Pst*. ^∗^*P* < 0.05. **(D)** Silencing efficiency in the *TaRac6*-knockdown plants inoculated with CYR31 were calculated using the 2^–ΔΔCT^ method. Three independent biological replications were performed to calculate the standard deviations and mean values. The wheat gene *TaEF-1*α was used to normalize the qRT-PCR data. Vertical bars represent the standard deviation. ^∗^*P* < 0.05.

**FIGURE 6:**
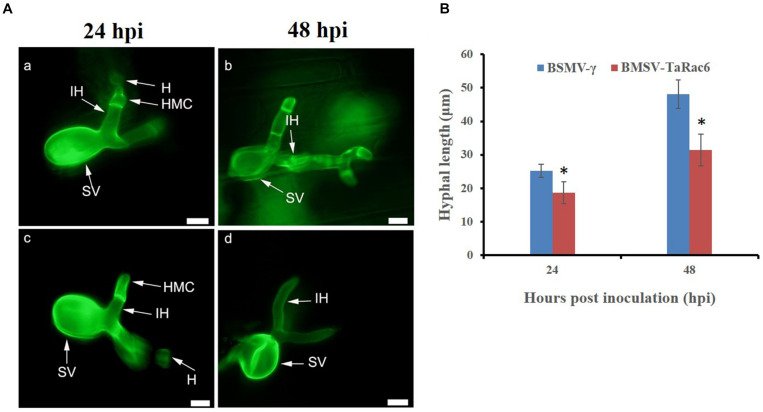
Histological observation of fungal growth in the *TaRac6*-knockdown plants inoculated with CYR31. **(A)** Microscopic observation of wheat pre-inoculated with BSMV-γ **(a,b)** and BSMV-*TaRac6* **(c,d)**. Wheat leaves inoculated with race CYR31 at 24 hpi and 48 hpi, respectively. The histological changes were stained with wheat germ agglutinin (WGA) and observed under a fluorescent microscope. H, haustoria; HMC, haustoria mother cell; IH, infection hypha; SV, substomatal vesicle. **(B)** Histological statistical analysis of hyphae lengths in the *TaRac6*-knockdown plants inoculated with CYR31 compared to BSMV-γ at 24 hpi and 48 hpi. The average distance was calculated from the substomatal vesicles to hyphal tips from 35 infection sites of three leaves. Means and standard deviations were calculated from three independent replicates. *P < 0.05. Bars = 10 μm.

**FIGURE 7:**
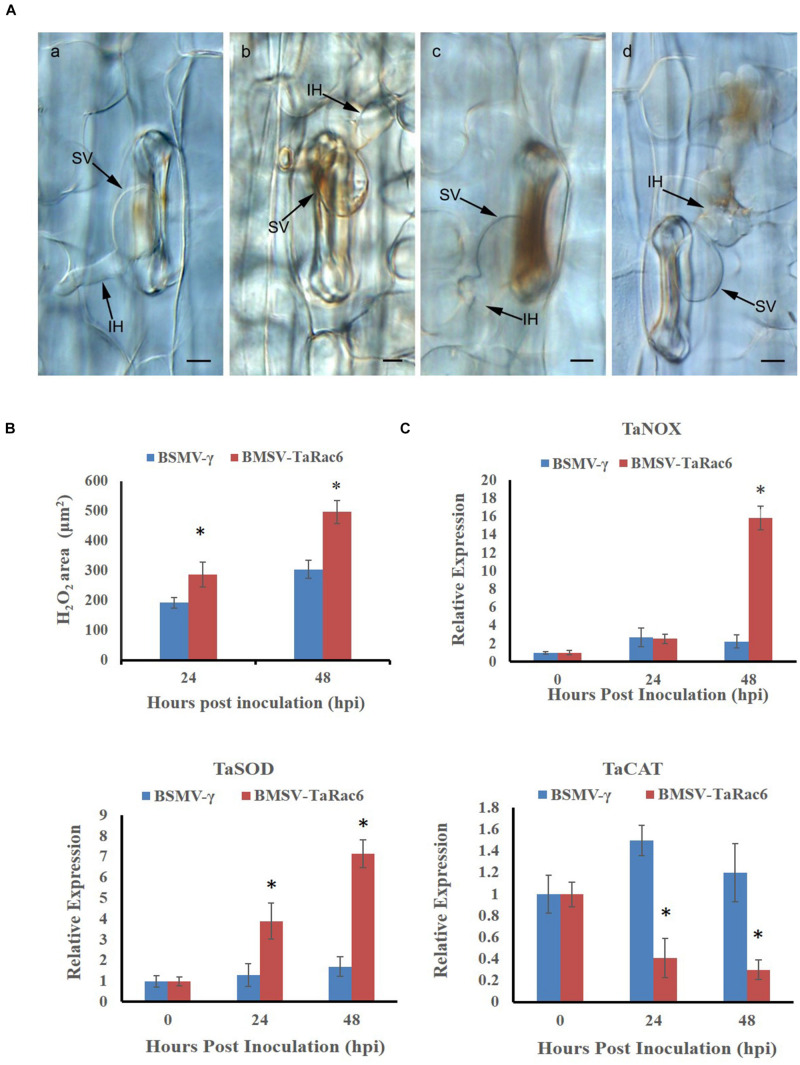
Histological observation of H_2_O_2_ accumulation by the 3, 3′-diaminobenzidine (DAB) staining. **(A)** (**a,b)** shows the histological of H_2_O_2_ accumulation in the control leaves at 24 hpi and 48 hpi, respectively; **(c,d)** shows the histological H_2_O_2_ accumulation in *TaRac6*-silenced leaves at 24 hpi and 48 hpi, respectively. IH, infection hypha; SV, substomatal vesicle. Bars = 10 μm. **(B)** Histological statistical analysis of H_2_O_2_ areas in the *TaRac6*-knockdown plants inoculated with CYR31 compared to BSMV-γ at 24 hpi or 48 hpi, respectively. The production of H_2_O_2_ was stained by the DAB and the average staining area was calculated at 35 infection sites. Statistical differences were assessed using Student’s *t* tests. *P < 0.05. **(C)** The qRT-PCR analysis of *TaCAL*, *TaSOD* and *TaNOX* in the *TaRac6*-knockdown plants inoculated with CYR31 using the 2^–ΔΔCT^ method. TaCAT, catalase (AKP21073.1); TaSOD, superoxide dismutase (JX398977.1). TaNOX, NADPH oxidase RBOHa (BK010636.1). BSMV-γ pre-inoculation plants served as the control. Three independent biological replications were performed to calculate the standard deviations and mean values. The wheat gene *TaEF-1*α was used to normalize the qRT-PCR data. Vertical bars represent the standard deviation. *P < 0.05.

The BSMV-VIGS system was used to silence the expression of *TaRac6* and thereby to characterize its function in the wheat–*Pst* interaction. Compared with the control leaves inoculated with FES-buffer, leaves inoculated with the vector of BSMV-γ and BSMV-TaRac6 displayed chlorotic striping at 10 days post-virus inoculation (dpvi) (Figures 5Aa–c). A bleaching phenotype was observed in PDS-silenced plants (Figures 5A,d), which suggested that the BSMV-VIGS system was effective. Race CYR31 was inoculated on the fourth leaf of wheat (Suwon 11), and 14 days later the leaves pre-inoculated with the FES-buffer and control leaves continued to display the typical compatible phenotype (Figures 5A,e,f); however, the susceptibility level of *TaRac6*-silenced leaves was significantly decreased (Figures 5A,g). The uredinia number in *TaRac6*-silenced leaves was reduced by approximately 30% relative to the control (BSMV-γ) (Figure 5B). Fungal DNA content was used as a proxy for *Pst* biomass in the leaves. The *Pst* DNA content was significantly reduced in *TaRac6*-silenced leaves indicating that fungal growth was inhibited (Figure 5C). To determine whether the phenotypic changes were caused by *TaRac6*’s silencing, qRT-PCR was used to detect the silencing efficiency compared with leaves inoculated with BSMV-γ. The transcript level of *TaRac6* was reduced by 67% and 71% at 24 hpi and 48 hpi, respectively (Figure 5D). This result indicated that the *TaRac6* gene had been successfully silenced. To determine whether the phenotypic changes between control and *TaRac6*-silenced plants are associated with fungal growth and development, *Pst* infection structures were stained and observed by microscopy. Histological analysis revealed a shorter hyphal length in *TaRac6*-silenced plants than that in control leaves (Figure 6). The accumulation of reactive oxygen species is considered to be the earliest inducing event in the plant-pathogens interaction, which controls and inhibits the growth of pathogens (Camejo et al., 2016). Therefore, to clarify whether the silencing of *TaRac6* led to the changes in host resistance level to *Pst*, DAB staining was used to detect the accumulation of H_2_O_2_ in leaves, the H_2_O_2_ accumulation area was significantly increased in *TaRac6*-silenced plants when compared with the control (BSMV-γ) at both 24 hpi and 48 hpi (Figures 7A,B).

To investigate the expression of genes known to control ROS accumulation, we selected *TaNOX*, *TaSOD*, and *TaCAT*. NOX enzymes are known to generate H_2_O_2_ (Lambeth, 2004), SOD catalyzes the conversion of superoxide anion to O_2_ and H_2_O_2_ (Fukai and Ushio-Fukai, 2011), and CAT is the major H_2_O_2_ scavenging enzyme (Yang and Poovaiah, 2002). To explore the reason of the H_2_O_2_ accumulation in *TaRac6*-silenced plants, qRT-PCR was used to analyze the transcript level of these ROS-related genes in comparison with the control plants. In the *TaRac6*-silenced plants, the expression of *TaSOD* and *TaNOX* increased compared with that in control leaves (BSMV-γ), while the transcript level of *TaCAT* was down-regulated in the *TaRac6*-knockdown wheat (Figure 7C). Altogether, the above results indicated that *TaRac6* might increase wheat susceptibility to *Pst* by inhibiting the production of H_2_O_2_.

## Discussion

The Rac/Rop signaling pathway has a significant role in regulating many organism activities (Takai et al., 2001). The Rac/Rop proteins of rice, *Medicago sativa*, and barley (*Hordeum vulgare*) are known to be critical for the establishment of those plants’ defense systems (Schiene et al., 2000; Schultheiss et al., 2003; Kawasaki et al., 2006; Chen et al., 2010). As in animals, such proteins can regulate the production of H_2_O_2_ by activating the NADPH oxidase at the plasma membrane (Park et al., 2000; Jones et al., 2007). Yet the function and mechanisms of similar Rac/Rop members in the response of plants to their pathogens remains largely understudied.

In this study, TaRac6 was isolated and characterized from wheat plants, and the involvement of *TaRac6* in wheat’s response to *Pst* was experimentally investigated. Sequence analysis showed that the TaRac6 contains a CxxL motif at its C-terminal. According to the C-terminal motif, Rac/Rop GTPases comprise two types. Type I proteins have a CxxL motif, while Type II possess a cysteine motif to anchor the membrane (Winge et al., 2000). On the basis of this protein domain, TaRac6 constituted a Type I protein, with further analysis showing that it occurred in the whole cell. In earlier work, Type II proteins of *A. thaliana* were found mainly localized at the plasma membrane (Lavy et al., 2002). Generally, however, unlike type II proteins, type I Rac/Rop proteins are more often detected in the whole cell, including its plasma membrane, cytoplasm, and nucleus (Chen et al., 2010).

Our phylogenetic analysis indicated that *TaRac6* encodes nearly the same amino acids as HvRacB and OsRac6. HvRacB was identified as a negative regulator of barley defense to *Bgh* (Schultheiss et al., 2003), and the expression of constitutively activated HvRacB made barley more susceptible to *Bgh* (Schultheiss et al., 2005; Pathuri et al., 2008). The RNAi lines of *HvRacB* markedly induced barley’s resistance to *Bgh* by restricting the formation of haustoria (Hoefle et al., 2011). Finally, overexpression of *OsRac6* enhanced susceptibility of rice to blast (Jung et al., 2006). Thus, in light of those findings, our results strongly suggest that TaRac6 is a potential susceptibility factor in wheat.

Bax-triggered plant cell death has similar physiological characteristics to plant HR. The cell death promoting function of Bax in plants correlated with accumulation of the defense-related protein PR1, suggesting that Bax activated an endogenous cell-death program in plants (Lacomme and Santa Cruz, 1999). This system has been used to successfully determine gene functioning as related to HR (Abramovitch et al., 2003; Wang et al., 2011). In our study, *TaRac6* was able to inhibit the cell death induced by Bax and the transcription levels of *PR1*α, *PR2*, and *PR5* were reduced in tobacco leaves when Bax was co-expressed with pGR106-TaRac6 or pGR106-Avr1b, compared to pGR106-eGFP, which indicated that TaRac6 could inhibit the Bax-triggered cell-death.

To define the potential role of *TaRac6* in the wheat–*Pst* interaction, qRT-PCR was used to detect the transcript level of *TaRac6*. This showed that *TaRac6* was highly induced in the compatible interaction, especially at 24 hpi, which is a critical time-point in the compatible interaction, marked by the formation of haustoria (Kang et al., 2002). The VIGS results also showed increased resistance when *TaRac6* was silenced. According to the results of histological observation and expression analysis of ROS-related enzymes, we speculated that silencing *TaRac6* drove an increase in H_2_O_2_ production. More ROS limited the normal expansion of hyphal at the infected sites, resulting in decreased sporulation. Thus, TaRac6 could affect the susceptibility of wheat to *Pst* by inhibiting the cell death triggered by the ROS burst. In rice and other plants, Rac proteins can regulate the production of H_2_O_2_ (Park et al., 2000; Jones et al., 2007).

As an important signaling molecule in plant cells, ROS is not only involved in programmed cell death, but also more importantly related to the formation of plant defense (Neill et al., 2002). In plants, Rac proteins have been shown to affect hydrogen peroxide production by regulating the activity of NADPH oxidase, which is necessary for the production of ROS (Bokoch and Diebold, 2002). Under hypoxic conditions, Rops are rapidly activated in *Arabidopsis*, resulting in ROP-dependent H_2_O_2_ production (Baxter-Burrell et al., 2002). In soybean cells, the Rac protein participated in the regulation of ROS production (Park et al., 2000). Overexpression of cotton GhRac13 promoted the production of H_2_O_2_ and then affected the formation of secondary walls of cotton cells (Potikha et al., 1999). It was speculated that Rac/Rops in dicotyledon may contribute to the ROS generation, however, the function of Rac/Rops to ROS generation in monocotyledon varies. In this study, TaRAC6 was demonstrated to play a negative role in wheat to *Pst* by inhibiting the H_2_O_2_ accumulation. Similarly, the mutant of *OsRac1* promotes ROS accumulation and cell death to increase the rice resistance (Kawasaki et al., 1999; Ono et al., 2001). The production of H_2_O_2_ mediated by CA-OsRac1 could be inhibited by DPI (NADPH oxidase inhibitor), indicating that NADPH oxidase downstream of OsRac1 regulated the production of H_2_O_2_ (Kawasaki et al., 1999). OsRac1 could interact with the NLR protein Pit to generate ROS and HR. The results showed that OsRac1 is required for Pit-mediated resistance to rice blast fungus (Kawano et al., 2010b). However, Rac genes in *Zea mays* could induce the production of ROS (Hassanain et al., 2000). What is more, constitutively activated mutant HvRacB has no significant effect on ROS production; it partially inhibited F-actin polarization distribution to *Bgh* invasion sites to prevent the invasion (Opalski et al., 2005). Another study further inferred that HvRacB could activate a ROP-binding protein kinase HvRBK1, which functioned in basal resistance to powdery mildew by affecting microtubule organization (Huesmann et al., 2012). In *Arabidopsis*, overexpression of *AtRac1* blocked the depolymerization of actin filaments during stomatal closure (Lemichez et al., 2001). In Barley, HvRacB was proven not only to regulate the reorganization of actin filaments to enhance susceptibility to *Bgh*, but also to affect stomata closure in ABA response (Schultheiss et al., 2005). Moreover, stomatal closure could be induced by ABA signaling in guard cells, which requires ROS formation to interact with Ca^2+^-channels (Kwak et al., 2006; Li et al., 2006). These results indicate that the plant Rac protein not only participates in regulating the production of ROS, but also affects the opening and closing of stoma. In this study, silencing of *TaRac6* could reduce ROS accumulation of wheat to improve the infection of *Pst*. As we know, *Pst* infects wheat leaves through stoma (Wang et al., 2007). Whether *TaRac6* plays a role in stomatal opening and closing and whether it affects the infection of *Pst* by affecting stomatal opening and closing needs be further explored.

## Conclusion

In conclusion, *TaRac6* was characterized in wheat’s response to *Pst*. As a type I Rac/Rop GTPase, TaRac6 was located in the whole cell, where it could inhibit the cell death induced by Bax. More importantly, *TaRac6* plays a role in governing the level of wheat susceptibility to *Pst* by affecting the ROS burst. This finding is of great significance in advancing the full functional exploration of Rac/Rop in plant responses to pathogens, and it lays a foundation for breeding disease-resistance in wheat by modifying its susceptibility genes.

## Data Availability Statement

The raw data supporting the conclusions of this article will be made available by the authors, without undue reservation, to any qualified researcher.

## Author Contributions

ZK and XW designed the experiments. QZ performed most of the experiments, analyzed the data, and wrote the manuscript. XZ, RZ, ZW, WS, XW, and ZK assisted in the experiments and discussed the results. All authors contributed to the article and approved the submitted version.

## Conflict of Interest

The authors declare that the research was conducted in the absence of any commercial or financial relationships that could be construed as a potential conflict of interest.

## Footnotes

1 https://www.ncbi.nlm.nih.gov

2 https://web.expasy.org/compute_pi/

3 http://www.cbs.dtu.dk/services/SignalP/

4 http://www.cbs.dtu.dk/services/TMHMM-2.0/#opennewwindow

5 https://wolfpsort.hgc.jp/

6 http://plants.ensembl.org/Triticum_aestivum/Info/Index

7 http://fungi.ensembl.org/Puccinia_graminis/Info/Index

## Supplementary Material

FIGURE S1 Multiple alignment of the cDNA sequence of the three copies of *TaRac6* isolated from wheat cultivar “Suwon11.”

FIGURE S2 Multiple alignment of the encoding sequence of the three copies of TaRac6.

FIGURE S3 Multiple alignment of TaRac6 against homologous amino acid sequences from other plant species. The red boxes indicate the G1–G5 boxes. The yellow box indicates the CxxL motif. Ta, *Triticum aestivum;* Os, *Oryza sativa*; Zm, *Zea mays*; At, *Arabidopsis thaliana*; Zm, *Zea mays*; Bd, *Brachypodium distachyon*. Different colors indicate homology levels of amino acids. The pink shading indicates at least 75% amino acid homology. Light blue shading indicates at least 50% amino acid homology.

FIGURE S4 The protein feature of TaRac6.

FIGURE S5 The original image file for western blot of TaRac6.
