# Supplementary figures and images for: TaRac6 Is a Potential Susceptibility Factor by Regulating the ROS Burst Negatively in the Wheat–Puccinia striiformis f. sp. tritici Interaction
Source: Front Plant Sci. 2020 Jun 30;11:716. doi: 10.3389/fpls.2020.00716 (PMC7338558; doi:10.3389/fpls.2020.00716)

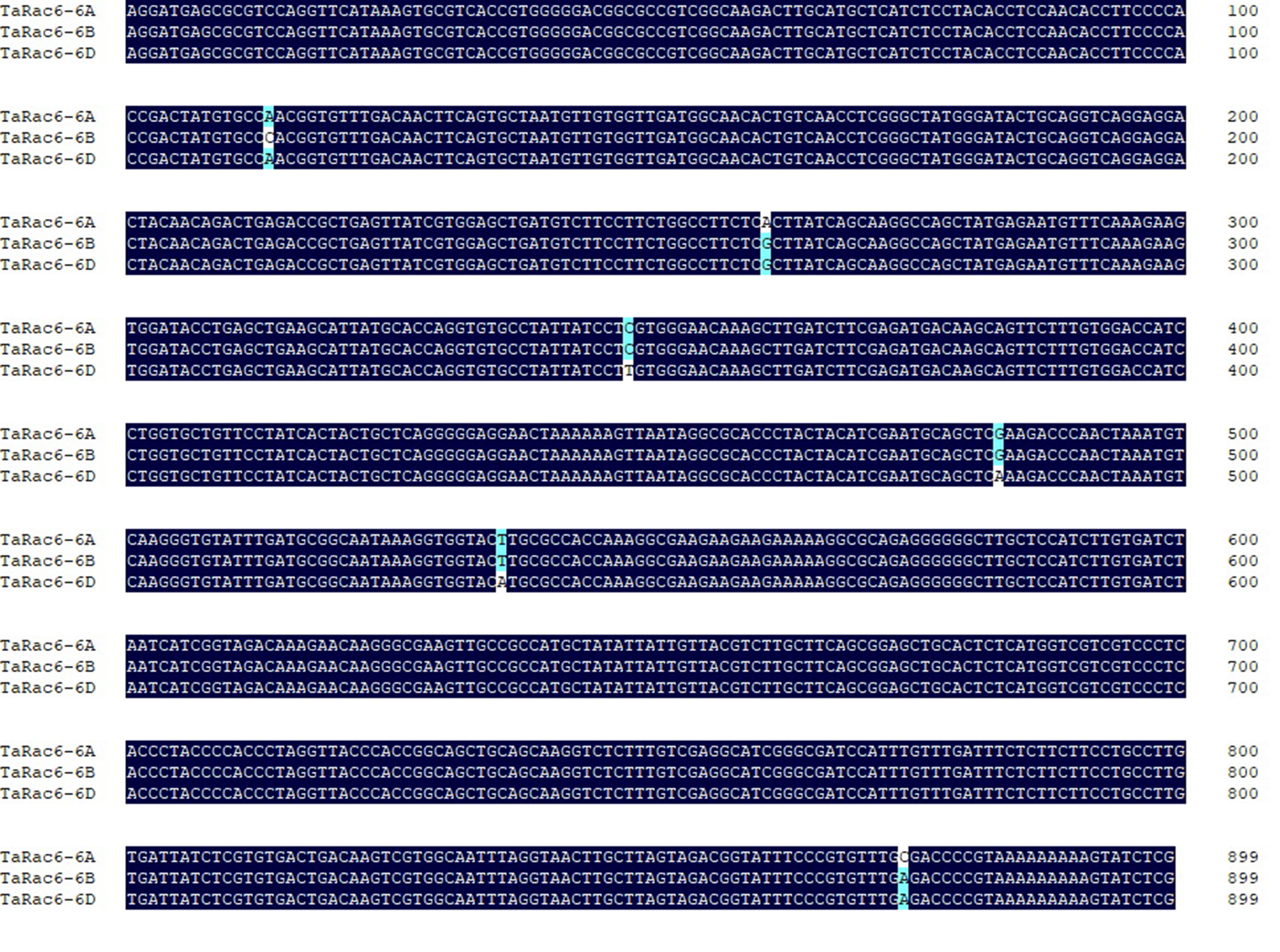

Supplement: FIGURE S1 — Multiple alignment of the cDNA sequence of the three copies of TaRac6 isolated from wheat cultivar “Suwon11.” [file Image_1.JPEG]

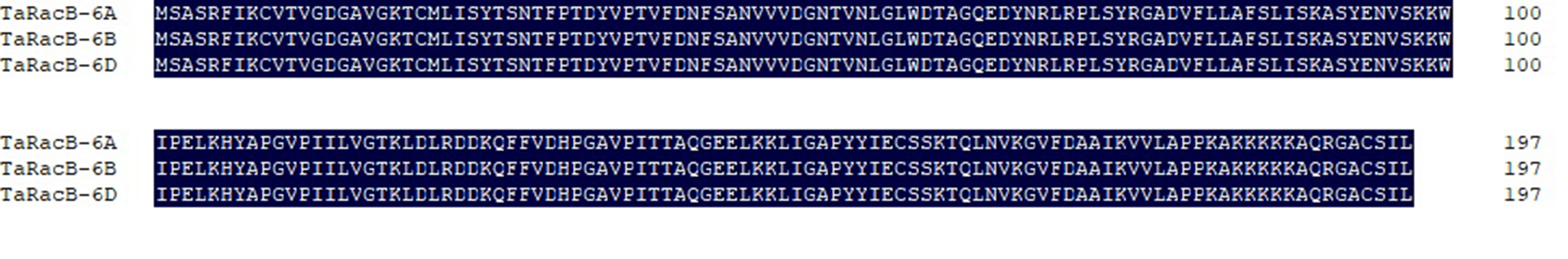

Supplement: FIGURE S2 — Multiple alignment of the encoding sequence of the three copies of TaRac6. [file Image_2.JPEG]

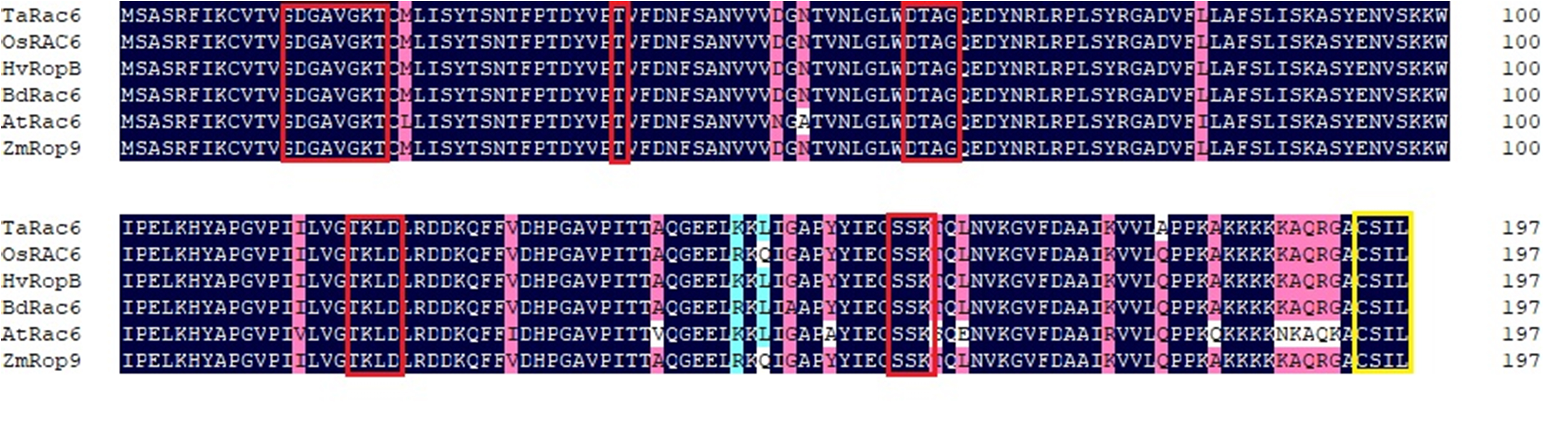

Supplement: FIGURE S3 — Multiple alignment of TaRac6 against homologous amino acid sequences from other plant species. The red boxes indicate the G1–G5 boxes. The yellow box indicates the CxxL motif. Ta, Triticum aestivum; Os, Oryza sativa; Zm, Zea mays; At, Arabidopsis thaliana; Zm, Zea mays; Bd, Brachypodium distachyon. Different colors indicate homology levels of amino acids. The pink shading indicates at least 75% amino acid homology. Light blue shading indicates at least 50% amino acid homology. [file Image_3.JPEG]

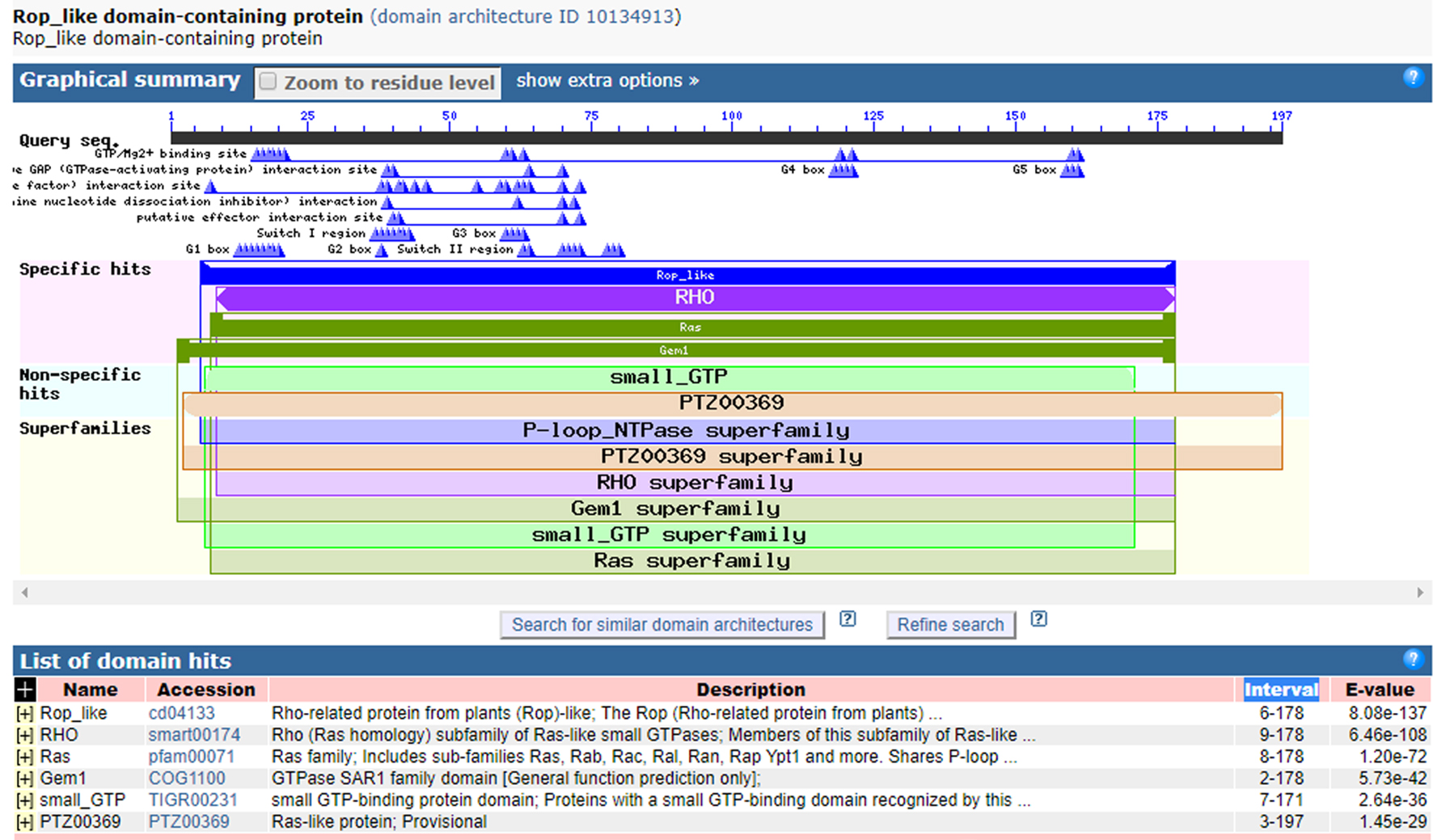

Supplement: FIGURE S4 — The protein feature of TaRac6. [file Image_4.JPEG]

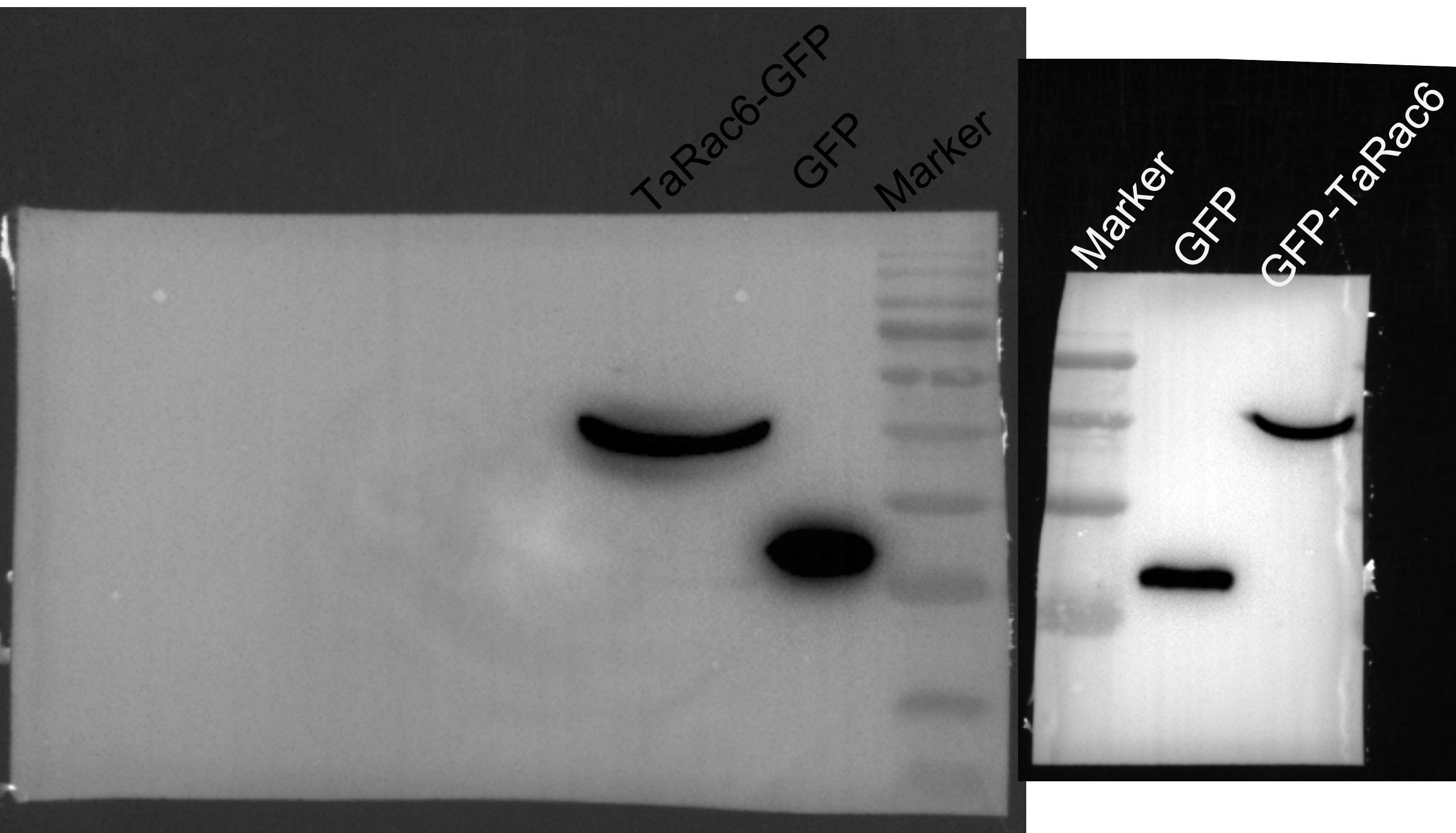

Supplement: FIGURE S5 — The original image file for western blot of TaRac6. [file Image_5.JPEG]
