# Supplementary material for: TaRac6 Is a Potential Susceptibility Factor by Regulating the ROS Burst Negatively in the Wheat–Puccinia striiformis f. sp. tritici Interaction
Source: Front Plant Sci. 2020 Jun 30;11:716. doi: 10.3389/fpls.2020.00716 (PMC7338558; doi:10.3389/fpls.2020.00716)
Supplement: Supplementary file 6 [file Table_1.docx]

**Table S1 | Primers used in this study**

| Name | Sequences |
| --- | --- |
| qRT-TaRac6-F | CAGTTCTTTGTGGACCATCC |
| qRT-TaRac6-R | GCGCAAGTACCACCTTTATT |
| qRT- TaEF-F | ACTCATGGTGCATCTCAACGGACT |
| qRT- TaEF-R | ACTCATGGTGCATCTCAACGGACT |
| TaRac6-GFP^C^-F | ATGGTAGATCTGACTAGT ATGAGCGCGTCCAGGTTC |
| TaRac6-GFP^C^-R  GFP^N^-TaRac6-F  GFP^N^-TaRac6-R | CACCATCCTAGGACTAGT CAAGATGGAGCAAGCCCC  GACGAGCTGTACAAGGGTACC ATGAGCGCGTCCAGGTTC  TCTAGTTCATCTAGAGGATCC CAAGATGGAGCAAGCCCC |
| pGR106-TaRac6-F | AGGTCAGCACCAGCTAGCATCGATATGAGCGCGTCCAGGTTC |
| pGR106-TaRac6-R | CTTAACCGTTCATCGGCGGTCGACCAAGATGGAGCAAGCCCC |
| VIGS-TaRac6-F | TAGCTAGCTGATTAATTAAAGGGGGAGGAACTAAAAAAG |
| VIGS-TaRac6-R | TTGCTAGCTGAGCGGCCGCACCGATGATTAGATCACAAG |
| qRT-TaPOD-F | TCCGTTGTCGCCTCTGGT |
| qRT-TaPOD-R | GTGCCTTGCCGATGGTGT |
| qRT-TaSOD-F | CCGAGGTCTGGAACCATCAC |
| qRT-TaSOD-R | AGCCGAAATCCTTCTCGATCT |
| qRT-TaNOX-F | ATGTTCGGCAACTTGGTGACT |
| qRT-TaNOX-R | CGTCTGCTCTAAGAAGACCACTTTT |
| qRT-NbActin-F | CCCAAAGGCTAATCGTGAAA |
| qRT-NbActin-R | ACCACGCTCTGTGAGGATCT |
| qRT-NbPR1α-F | CGACCAGGTAGCAGCCTATG |
| qRT-NbPR1α-R | ACCCTAGCACATCCAACACG |
| qRT-NbPR2-F | CCTTCCACTCTTAGCCAATGTCTATCC |
| qRT-NbPR2-R | AGAAGGCCAGCCACTTTCAGATAC |
| qRT-NbPR5-F | CCGAGGTAATTGTGAGACTGGAG |
| qRT-NbPR5-R | CCTGATTGGGTTGATTAAGTGCA |
